# A Maturation-Aware Machine Learning Framework for Screening the Nutritional Status of Adolescents

**DOI:** 10.3390/nu18040660

**Published:** 2026-02-17

**Authors:** Hatem Ghouili, Zouhaier Farhani, Narimen Yousfi, Halil İbrahim Ceylan, Amel Dridi, Andrea de Giorgio, Nicola Luigi Bragazzi, Noomen Guelmami, Ismail Dergaa, Anissa Bouassida

**Affiliations:** 1 Research Unit: Sports Science, Health and Movement, High Institute of Sport and Physical Education of Kef, University of Jendouba, El Kef 7100, Tunisia; hatemghouili@gmail.com (H.G.); zouhaierfarhani@gmail.com (Z.F.); yousfinarimen@gmail.com (N.Y.); ameldridi291@gmail.com (A.D.); noomenstat@gmail.com (N.G.); anissa.bouassida@gmail.com (A.B.); 2 Physical Education and Sports Teaching Department, Faculty of Sports Sciences, Atatürk University, Erzurum 25240, Türkiye; 3 Artificial Engineering, 80121 Naples, Italy; 4 Laboratory for Industrial and Applied Mathematics (LIAM), Department of Mathematics and Statistics, York University, Toronto, ON M3J 1P3, Canada; 5 Human Nutrition Unit (HNU), Department of Food and Drugs, University of Parma, 43121 Parma, Italy; 6 Department of Clinical Pharmacy, Saarland University, 66123 Saarbrücken, Germany; 7 Higher Institute of Sport and Physical Education of Ksar Saïd, University of Manouba, Manouba 2010, Tunisia; phd.dergaa@gmail.com; 8 Physical Activity Research Unit, Sport and Health (UR18JS01), National Observatory of Sports, Tunis 1003, Tunisia

**Keywords:** adolescent nutrition, biological maturation, class imbalance, machine learning, peak height velocity, random forest, underweight

## Abstract

**Background:** Malnutrition in adolescents remains a significant public health issue worldwide, with undernutrition and overweight often coexisting. Accurate nutritional screening during adolescence is complicated by variability in biological maturation and class imbalance, particularly among underweight adolescents. **Objective:** This study aims to develop and validate machine learning models for classifying the nutritional status of adolescents, accounting for class imbalance and biological maturation, and to evaluate model stability and variable importance at different stages of peak height velocity (PHV). **Methods:** In this cross-sectional study, 4232 adolescents aged 11 to 18 years were recruited from nine educational institutions in Tunisia. Their nutritional status was classified according to the International Obesity Task Force (IOTF) BMI thresholds into three categories: underweight (14.4%), normal weight (68.3%), and overweight (17.2%). Ten anthropometric, behavioral, and maturation-related predictors were analyzed. Six supervised machine learning algorithms were evaluated using a 70/30 stratified split between training and test sets, with five-fold cross-validation. Class imbalance was addressed by ROSE combined with cost-sensitive learning. Model performance was assessed using accuracy, Cohen’s kappa coefficient, macro F1 score, sensitivity, specificity, and AUC. **Results:** The cost-sensitive Random Forest (RF) model achieved the best overall performance, with an accuracy of 0.830, a macro F1 score of 0.767, a macro-AUC of 0.921, and a macro- sensitivity of 0.743. The class-specific sensitivities were 0.70 (underweight), 0.91 (normal weight), and 0.62 (overweight), with no major misclassification between the extreme categories. Performance remained stable across the different maturation phases (accuracy from 0.823 to 0.839), with optimal discrimination in the pre-PHV (macro-AUC = 0.936; sensitivity for underweight = 0.82) and post-PHV (macro-AUC = 0.931) periods. Body mass was the main predictor (importance = 1.00), followed by waist circumference (0.34–0.53). The importance of age for classifying underweight increased significantly from the pre-PHV (0.10) to the post-PHV (0.75) period. A two-stage hierarchical model further improved underweight detection (stage 1 AUC = 0.911; sensitivity = 0.732). **Conclusions:** A cost-sensitive RF model, combined with ROSE, provides robust classification of adolescents’ nutritional status maturation, significantly improving underweight detection while preserving overall accuracy. This approach is particularly well-suited to public health screening in schools as a first-stage assessment that requires clinical confirmation and promotes a maturation-aware interpretation of nutritional risk among adolescents.

## 1. Introduction

Child and adolescent malnutrition present a significant developmental risk, with serious long-term health consequences. In 2024, 150.2 million children under five years experienced stunting, 42.8 million suffered from wasting, and 35.5 million were affected by obesity worldwide [1]. Additionally, over 390 million children and adolescents aged 5 to 19 years were overweight in 2022, including approximately 160 million with obesity [2]. This double burden disproportionately affects low- and middle-income countries (LMICs), where undernutrition and excess adiposity coexist within populations [3,4]. In fact, adolescence is a period of increased nutritional requirements due to rapid linear growth, sexual maturation, and substantial increases in lean body mass and bone mineral density. Nutritional inadequacy during this period compromises pubertal progression, peak bone mass attainment, neurocognitive development, and immunological competence [5,6,7]. Conversely, excess energy intake and adiposity during adolescence establish metabolic programming that predicts adult cardiovascular disease, type 2 diabetes, and obesity-associated malignancies [8,9]. Adolescent growth is affected by substantial inter-individual variation in maturation timing, spanning approximately four years between early and late maturation, which creates challenges for nutritional assessment and risk stratification [7,10,11].

Traditional nutritional screening relies primarily on body mass index (BMI) classification based on age- and sex-specific reference standards. The International Obesity Task Force (IOTF) developed Body Mass Index (BMI) cut-offs to improve international comparability by analyzing six large, nationally representative datasets and linking adolescent thresholds to adult BMI values of 18.5, 25, and 30 kg/m^2^ [12,13]. These classifications provide standardized approaches but do not account for heterogeneity in biological maturation. Peak height velocity (PHV), the period of maximum growth rate during puberty, varies significantly in timing and magnitude among individuals [14,15]. Early-maturing adolescents enter rapid growth approximately two years earlier than late-maturing peers, resulting in substantial overlap in anthropometric distributions across chronological ages. This maturational heterogeneity increases the risk of misclassification when nutritional status is assessed using chronological age-based thresholds that do not account for maturation [16,17]. The multifactorial nature of adolescent nutritional status and its interaction with the timing of biological maturation suggests potential advantages for multivariable predictive approaches that account for maturation-specific patterns. Research suggests that physical activity, sleep patterns, psychosocial stress exposure, and dietary behaviors follow distinct developmental trajectories during adolescence, with varying impacts on energy balance across maturation stages [18,19]. To improve classification precision, Machine Learning (ML) algorithms provide a tool for integrating heterogeneous variables, such as anthropometrics, behavior, and maturation [20,21]. In this area, supervised classification methods, including random forests (RFs), support vector machines (SVMs), and gradient boosting (XGBoost), have demonstrated effectiveness in predicting pediatric obesity, identifying metabolic syndrome, and stratifying cardiovascular risk [21,22,23].

Despite methodological advances in ML and increased recognition of the influence of maturation on adolescent health, significant knowledge gaps persist. No studies have systematically examined whether nutritional status classification algorithms maintain stable performance across PHV stages, or how the importance of anthropometric and behavioral predictors changes during maturation. The interaction between biological maturation timing and classification accuracy remains uncharacterized, as does the practical application of class imbalance correction specifically for adolescent underweight detection. These gaps hinder the development of maturation-informed screening approaches that could improve early identification of at-risk adolescents while accounting for developmental stage-specific factors.

Therefore, our study aimed to (i) develop and validate six ML algorithms to classify adolescent nutritional status as underweight, normal weight, or overweight using anthropometric, behavioral, and maturational predictors; (ii) identify optimal predictive performance through comprehensive evaluation, accounting for underweight minority class imbalance correction; (iii) assess classification accuracy stability across pre-PHV, during-PHV, and post-PHV biological maturation stages; and (iv) characterize how variable importance for nutritional status classification shifts across maturation phases.

## 2. Materials and Methods

### 2.1. Ethical Approval and Artificial Intelligence Usage

This investigation received ethical approval from the Institutional Scientific and Ethical Committee for Physical Education and Sports (ISSEP El Kef), University of Jendouba, Tunisia, under reference PHS-32/2022 (date: 27 January 2022). It also complied with the ethical and procedural requirements for conducting sports medicine and exercise science research [24]. Written informed consent was obtained from the parents or legal guardians of all participants before enrolment. Adolescents provided written assent after an age-appropriate explanation of the study procedures, data collection methods, and confidentiality protection. The study protocol adhered to the principles of the Declaration of Helsinki for human research.

During the preparation of this work, the authors used ChatGPT version 5.1 (OpenAI, San Francisco, CA, USA) on 9 December 2025 to revise specific passages and assess grammatical accuracy and academic English quality [25,26]. All scientific content, data analysis, interpretation, and conclusions were developed independently by the authors. After using the tool, all content was critically reviewed and corrected by the authors, who assumed full responsibility for the manuscript.

### 2.2. Sample Size Calculation

Sample size calculation used the formula *n* = Z^2^*p*(1 − *p*)/d^2^, where Z is the standard normal variate (1.96 for a 95% confidence interval or CI), *p* is the expected proportion (0.15 for underweight prevalence), and d is the desired precision (0.02). The calculation yielded *n* = (1.96)^2^(0.15)(0.85)/(0.02)^2^ = 1224 participants as the minimum requirement. Power analysis for multinomial logistic regression (MLR) with three outcome categories was performed using G*Power version 3.1.9.7 (Heinrich Heine University Düsseldorf, Düsseldorf, Germany), specifying a medium effect size (Cohen’s w = 0.3), a Type I error probability α = 0.05, and a statistical power of 1 − β = 0.80. This analysis indicated a minimum sample of 1089 participants. Previous cross-sectional studies of adolescent nutritional status in North African populations enrolled samples ranging from 2847 to 5612 participants and reported small to medium effect sizes [27,28]. The final sample of 4232 adolescents substantially exceeded calculated requirements and provided adequate statistical power for stratified analyses across three maturation stages and three nutritional status categories.

### 2.3. Participants

Participants were recruited from nine educational institutions in Tunisia, including two primary schools (grades 5 and 6), four preparatory schools, and three secondary schools. Recruitment took place between January 2022 and March 2025 through coordination with school administrations and physical education departments. Inclusion criteria were as follows: (i) chronological age 11 to 18 years, (ii) active enrolment during the assessment period, and (iii) complete anthropometric measurements and questionnaire responses for all predictor variables. Exclusion criteria were as follows: (i) the presence of chronic diseases with potential impact on nutritional status or growth trajectories (e.g., chronic inflammatory, renal, gastrointestinal, or systemic diseases), (ii) chronic medication use known to influence body composition, including corticosteroids, antipsychotics, or anticonvulsants, (iii) physical disabilities precluding anthropometric measurement, and (iv) incomplete data for any predictor variable. Of the 4458 adolescents initially approached, 4232 met the inclusion criteria and provided complete data, yielding a 95% completion rate. Trained field researchers conducted all assessments according to standardized protocols, with prior certification in anthropometric techniques.

### 2.4. Experimental Design

This cross-sectional investigation used systematic assessment protocols during regular school hours. All measurements were taken in private rooms to maintain participant confidentiality. Anthropometric assessments were conducted between 08:00 and 11:00 to minimize the effects of diurnal variation. Participants wore light indoor clothing and no footwear during measurements. Each anthropometric parameter was measured twice, with a third measurement taken if the initial values differed by more than 0.5 cm for height or 0.2 kg for body mass. Mean values were used for analysis.

Body mass was measured using calibrated electronic scales (Seca 813, Hamburg, Germany) with a precision of 0.1 kg. Standing height was assessed using portable stadiometers (Seca 213, Hamburg, Germany) with 0.1 cm precision, in accordance with standard anthropometric protocols [29]. Sitting height was measured with participants seated on standardized height boxes (50 cm high), backs straight, knees at 90 degrees, feet flat on the floor, and hands resting on the thighs. Leg length was calculated as standing height minus sitting height. Waist circumference was measured at the narrowest point between the lowest rib margin and the iliac crest at end-expiration, using non-stretch measuring tape (Seca 201, Hamburg, Germany) with 0.1 cm precision, following the World Health Organization (WHO) protocols [30]. The tape was positioned horizontally, maintained in firm contact without compression, and measurements were recorded to the nearest 0.1 cm.

BMI was calculated as weight (kg) divided by height squared (m^2^). Nutritional status was classified using IOTF age-specific and sex-specific BMI cut-offs, with exact age calculated in months from birth date to assessment date. Classifications served as screening indicators requiring clinical confirmation rather than definitive diagnosis. Underweight was defined as a BMI below the IOTF cut-off corresponding to an adult BMI of 18.5 kg/m^2^. Normal weight included values between the underweight and overweight thresholds. Overweight was defined as a BMI exceeding the IOTF cut-off corresponding to an adult BMI of 25 kg/m^2^ [12,13].

### 2.5. Assessment Instruments

#### 2.5.1. Physical Activity Assessment

Physical activity was assessed using age-appropriate validated questionnaires. The Physical Activity Questionnaire for Children (PAQ-C) was administered to participants aged 11–13 years, and the Physical Activity Questionnaire for Adolescents (PAQ-A) to those aged 14–18 years [31,32]. These instruments assess general physical activity levels using nine items that evaluate activities during physical education classes, recess, lunch periods, after-school hours, evenings, and weekends. The Arabic-validated versions demonstrated acceptable internal consistency (Cronbach’s α = 0.70–0.81) and construct validity [33]. Composite scores were calculated as arithmetic means of items rated on 5-point scales, with higher values indicating greater physical activity engagement [34,35].

#### 2.5.2. Sleep Duration Assessment

Sleep duration was assessed using items from the Adolescent Sleep Questionnaire [36]. Participants reported their typical bedtimes and wake times separately for weekdays and weekends. Average nightly sleep duration was calculated as: [(weekday sleep duration × 5) + (weekend sleep duration × 2)]/7, expressed in hours per night. Sleep duration values below 4 h or above 12 h were flagged for verification to identify potential reporting errors.

#### 2.5.3. Perceived Stress Assessment

Perceived stress was measured using the 10-item Perceived Stress Scale, which assesses the degree to which situations during the past month were appraised as stressful [37]. The Arabic adaptation demonstrated acceptable internal consistency (Cronbach’s α = 0.74–0.77) [38]. Items were rated on 5-point Likert scales from 0 (never) to 4 (very often). Four positively worded items (items 4, 5, 7, 8) were reverse-scored before summing. Total scores ranged from 0 to 40, with higher values indicating greater perceived stress.

#### 2.5.4. Dietary Diversity Assessment

Dietary diversity was assessed using a food-group-based dietary diversity score, adapted from the Food and Agriculture Organization of the United Nations (FAO) recommendations [39]. Participants reported their consumption of foods from ten predefined food groups over the previous 24 h using a standardized questionnaire administered by trained interviewers. Each food group contributed 1 point to the total dietary diversity score, yielding a possible score of 0–10, with higher values indicating greater dietary diversity.

This score was used as an indirect indicator of overall diet quality at the population level, reflecting the variety of food groups consumed rather than total energy intake or macronutrient composition. The dietary diversity score was included as a continuous predictor variable in the ML models.

#### 2.5.5. Biological Maturation Assessment

Biological maturity was assessed using PHV, defined as the period of maximum linear growth rate in height, which occurs during puberty and represents a milestone in physical maturation. PHV indicates the timing of the growth spurt during adolescence and varies considerably between individuals, regardless of chronological age.

Biological maturation was estimated using the Mirwald sex-specific maturity offset equations, which predict years to PHV from anthropometric measurements [40]. The equations include chronological age, height, sitting height, leg length, and their statistical interactions.

For males: Maturity Offset = −9.236 + 0.0002708 (Leg Length × Sitting Height) − 0.001663 (Age × Leg Length) + 0.007216 (Age × Sitting Height) + 0.02292 (Weight by Height ratio).

For females: Maturity Offset = −9.376 + 0.0001882 (Leg Length × Sitting Height) + 0.0022 (Age × Leg Length) + 0.005841 (Age × Sitting Height) − 0.002658 (Age × Weight) + 0.07693 (Weight by Height ratio).

Maturity offset values represent estimated years before (negative values) or after (positive values) the occurrence of PHV. Participants were categorized into three maturation stages: pre-PHV (maturity offset less than −1.0 years), during-PHV (maturity offset −1.0 to +1.0 years), and post-PHV (maturity offset greater than +1.0 years). These categories enabled performance evaluation and variable importance analysis stratified by biological maturation phase.

### 2.6. Statistical Analysis

All analyses were performed using R software version 4.3 (R Foundation for Statistical Computing, Vienna, Austria). Descriptive statistics summarized the demographic characteristics, anthropometric measurements, and behavioral variables of participants by nutritional status. Continuous and categorical variables were presented as means ± standard deviations and percentages, respectively. Differences between groups were evaluated using a one-way analysis of variance (ANOVA) with Tukey’s post hoc tests and chi-square tests for continuous and categorical variables, respectively. The threshold for statistical significance was set at *p* < 0.05.

The dataset was randomly divided into a training set (70%) and an independent test set (30%) using stratified sampling. All model development and optimization procedures were performed on the training set, while performance evaluation was performed on the test set. Six supervised ML algorithms were implemented to classify adolescents according to their weight (underweight, normal weight, or overweight): RF, decision tree (DT), k-nearest neighbors (k-NN), SVM with radial basis function kernel, MLR, and eXtreme Gradient Boosting (XGBoost). The models were trained using five-fold cross-validation. Hyperparameters were optimized for RF and XGBoost on the training set, while standard optimization strategies were applied to the other algorithms. Centering and normalization of features were applied to distance-based models, while tree-based models were trained on unnormalized data. Class imbalance was addressed using the ROSE (Random Oversampling Examples) algorithm [41,42,43] to improve the representation of minority classes and model sensitivity. Detailed descriptions of procedures are provided in the Supplementary Materials. The model’s performance was evaluated using precision, Cohen’s kappa coefficient, and the Receiver Operating Characteristic (ROC) curve (micro- and macro-averaged Area Under the Curve (AUC) metrics). Confusion matrices were used to visualize and examine error patterns. Variable importances were assessed using both mean decrease in Gini impurity and permutation importance to verify consistency across methods. Permutation importance results showed highly consistent variable rankings with Gini-based measures (rank correlation exceeding 0.85), suggesting that methodological bias did not substantially affect our conclusions [44,45,46]. In addition, variable importance analyses were performed separately for each nutritional category (underweight, normal weight, and overweight) and for each stage of biological maturation (pre-PHV, during PHV, and post-PHV).

## 3. Results

### 3.1. Participant Characteristics

The sample comprised 4232 adolescents, including 610 underweight (14.4%), 2893 normal-weight (68.3%), and 729 overweight (17.2%) individuals (Table 1). The mean age was 13.76 ± 2.25 years (range: 11–18 years), with a balanced sex distribution (49.9% girls and 50.1% boys).

- ***Anthropometric Characteristics***

Body mass and waist circumference increased significantly across nutritional categories (*p* < 0.001 for both). Mean body mass rose from 37.1 kg in underweight adolescents to 48.5 kg in those of normal weight and to 60.9 kg in those who were overweight, while waist circumference increased from 65.5 cm to 70.6 cm and then to 77.1 cm.

Standing height differed slightly between groups (*p* < 0.001), with underweight adolescents being slightly shorter than their normal-weight peers. Overweight adolescents had a slightly shorter sitting height and shorter legs than the other groups (*p* < 0.05), suggesting proportionally shorter limbs relative to trunk length, a pattern consistent with earlier biological maturation in adolescents with excess adipose tissue.

- ***Behavioral Characteristics***

Sleep duration varied significantly by nutritional status (*p* < 0.001), with underweight adolescents reporting the shortest sleep duration (6.5 ± 0.8 h/night), followed by normal-weight (6.8 ± 0.8 h) and overweight (7.2 ± 0.9 h) adolescents. Physical activity showed the opposite pattern (*p* < 0.001), with underweight adolescents being the most active and overweight adolescents the least active.

Perceived stress increased progressively across nutritional categories (*p* < 0.001), from underweight to overweight adolescents. In contrast, dietary diversity did not differ significantly between the groups (*p* = 0.41).

- ***Sex and Biological Maturation Distribution***

Sex distribution differed slightly across nutritional categories (*p* = 0.043). Girls represented approximately half of both underweight and normal-weight adolescents, but a smaller proportion of overweight participants.

The distribution of biological maturation stages differed significantly according to nutritional status (*p* < 0.001). Underweight adolescents were more frequently classified as pre-PHV (pre-pubertal growth phase), while normal-weight adolescents were predominantly in the pubertal growth phase. Overweight adolescents had a higher proportion of pre-PHV than their normal-weight peers, with similar proportions of post-PHV across all nutritional categories.

**Table 1:** Participant Characteristics by Nutritional Status. Abbreviations: PHV (Peak Height Velocity); SD (Standard Deviation).

Variable	Underweight (*n* = 610)	Normal (*n* = 2893)	Overweight (*n* = 729)	*p*-Value
Age, years (mean ± SD)	13.98 (2.61)	13.70 (2.13)	13.78 (2.46)	0.022
Body mass, kg (mean ± SD)	37.06 (9.80)	48.50 (11.06)	60.92 (15.22)	<0.001
Height (cm)	155.93 (14.58)	158.08 (12.10)	156.56 (12.73)	<0.001
Waist circumference, cm (mean ± SD)	65.53 (8.53)	70.57 (7.52)	77.11 (10.43)	<0.001
Sitting height, cm (mean ± SD)	77.14 (7.93)	77.61 (7.45)	76.89 (7.29)	0.041
Lower Limb	78.73 (11.03)	80.47 (9.49)	79.62 (9.99)	<0.001
Sleep duration, h/day (mean ± SD)	6.53 (0.76)	6.82 (0.81)	7.15 (0.94)	<0.001
Physical activity score	3.27 (1.12)	2.85 (0.88)	2.66 (0.77)	<0.001
Stress score	16.35 (7.17)	18.64 (6.08)	20.87 (6.01)	<0.001
Dietary diversity score	4.49 (2.62)	4.37 (2.80)	4.28 (2.87)	0.410
Girls, *n* (%)	310 (50.8%)	1469 (50.8%)	333 (45.7%)	0.043
PHV Stage, *n* (%)				<0.001
• Pre-PHV	227 (22.5%)	552 (54.7%)	231 (22.9%)	
• During-PHV	147 (9.4%)	1202 (77.0%)	212 (13.6%)	
• Post-PHV	236 (14.2%)	1139 (68.6%)	286 (17.2%)	

Continuous variables: mean ± SD, ANOVA *p*-values. Categorical variables: *n* (%), χ^2^ test.

### 3.2. Machine Learning Model Performance

Table 2 presents the performance of all ML models evaluated on the independent test set (*n* = 1268). Overall accuracy ranged from 0.760 to 0.830.

The cost-sensitive RF model with oversampling (ROSE) consistently outperformed all other models across both overall and class-balanced metrics. It achieved the highest accuracy (0.830), the highest Cohen’s kappa coefficient (0.629), the highest macro F1 score (0.767), the best macro sensitivity (0.743), the best macro specificity (0.861), and the best macro AUC value (0.921), as well as strong overall discrimination (micro-AUC = 0.898). Therefore, this model was selected as the optimal classifier for subsequent analyses (Table 2).

Other algorithms, including SVM, MLR, k-NN, DT, and XGBoost, exhibited lower performance and greater heterogeneity. While some models achieved acceptable overall discrimination (macro-AUC ≈ 0.81–0.90), they consistently showed poor agreement (kappa ≤ 0.616) and inferior performance regarding class balance, particularly for underweight adolescents. Despite high AUC values, XGBoost demonstrated the weakest performance for the minority class, with low sensitivity for underweight, underscoring the importance of modeling strategies that address class imbalance (Table 2).

**Figure 1:**
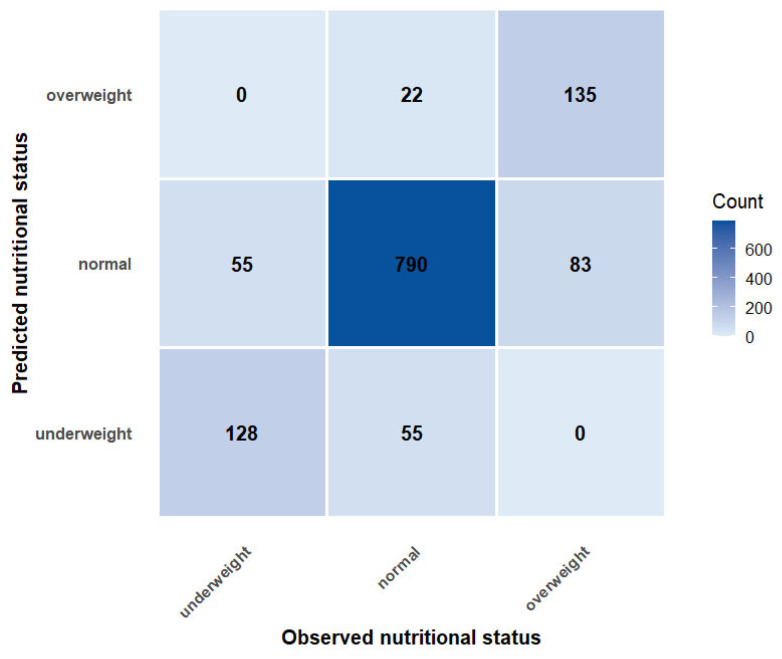
Confusion Matrix of Cost-Sensitive Random Forest.

Analysis of the confusion matrix (Figure 1) provided a clearer understanding of the class-specific behavior of the optimal RF model. The matrix revealed strong diagonal dominance, indicating high overall classification accuracy, with misclassifications occurring almost exclusively between adjacent nutritional categories. Normal-weight adolescents were classified with the highest accuracy, with 790 out of 867 individuals correctly identified (sensitivity = 0.91). Underweight adolescents were identified with acceptable sensitivity (128 out of 183; 0.70), with all misclassifications shifting towards the “normal weight” category. Classification of overweight adolescents demonstrated moderate sensitivity (135 of 218; 0.62), with errors limited to misclassification of normal-weight individuals. Importantly, no misclassifications were observed between the extreme categories (underweight and overweight), highlighting the clinical robustness and stability of the model’s decision boundaries.

### 3.3. Performance Across Biological Maturation Stages

Table 3 summarizes the performance of the cost-sensitive RF model, stratified by PHV stage. Overall accuracy remained consistently high throughout the maturation phases, with values of 0.827 in pre-PHV adolescents, 0.823 during-PHV, and 0.839 post-PHV. Cohen’s kappa coefficients indicated moderate to substantial agreement, with the highest value observed in pre-PHV (0.701), followed by post-PHV (0.646) and during-PHV (0.498) (Table 3).

The model’s discrimination was strongest at the extremes of the biological maturation spectrum. Pre-PHV adolescents demonstrated excellent performance, with a macro F1 score of 0.814, a macro sensitivity of 0.815, and the highest macro-AUC (0.936). This indicates particularly effective detection of underweight among early adolescents (sensitivity to underweight = 0.82). Similarly, adolescents in the post-PHV period demonstrated excellent classification performance, with an overall F1 score of 0.777, an overall sensitivity of 0.760, an overall specificity of 0.870, and an overall AUC of 0.931 (Table 3).

In contrast, the PHV period is the most challenging classification context. Although overall accuracy remained acceptable (0.823), balanced classification performance declined, with an overall F1 score of 0.674 and an overall sensitivity of 0.623. The class analysis revealed reduced sensitivity for underweight (0.58) and overweight (0.41) adolescents, whereas the classification of normal-weight adolescents remained high (0.89), indicating increased overlap in nutritional phenotypes during the pubertal transition (Table 3).

### 3.4. Variable Importance Across Maturation and Nutritional Status

**Figure 2:**
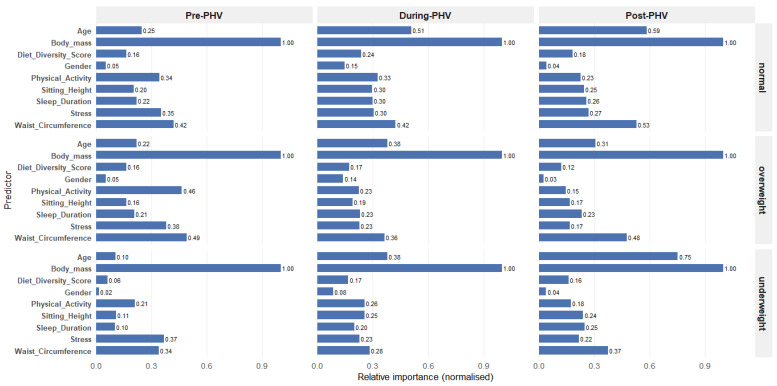
Variable importance analysis of each nutritional status across the PHV stage.

Figure 2 shows the normalized variable importance profiles for each nutritional status category across the different stages of PHV. Body mass was the primary predictor in all models, consistently achieving the highest variable importance (normalized value = 1.00).

Waist circumference ranked second for all nutritional categories and maturation stages, with stable variable importance values (0.34–0.53), indicating a consistent contribution throughout adolescence. In contrast, chronological age showed marked stage-dependent effects, particularly for the underweight classification, where variable importance increased sharply from 0.10 pre-PHV to 0.75 post-PHV. A similar but less pronounced increase was observed for the normal-weight classification (0.25–0.59), whereas the variable importance of age for the overweight classification remained relatively stable (0.18–0.24).

The other predictors showed modest and category-specific contributions. Sitting height demonstrated moderate and stable variable importance (0.15–0.28). Physical activity contributed moderately, with a slightly greater effect for the underweight classification (0.12–0.24), whereas sleep duration showed a lower and more variable effect (0.08–0.19). Perceived stress had a greater influence on overweight classification (0.28–0.38) than on underweight or normal-weight models. Dietary diversity and sex consistently showed minimal variable importance (≤0.12 and 0.14, respectively).

## 4. Discussion

The present study developed and validated an ML framework to classify adolescents’ nutritional status, addressing class imbalance and the effects of biological maturation. Of the six algorithms evaluated, a cost-sensitive RF model combined with ROSE achieved the best class balance, consistent with previous findings supporting ensemble methods for clinical prediction tasks [20,23,43]. Notably, classification accuracy remained stable across the PHV stages, confirming the applicability of this approach throughout the adolescent growth period [7,10,14]. Moreover, variable importance analyses revealed biologically relevant and maturation-dependent patterns, providing insights into adolescent growth dynamics that are useful for screening in the general population.

In line with previous research, RF algorithms are well-suited to nutritional data owing to their robustness to nonlinear relationships, variable interactions, and mixed data types [44,47,48]. However, the classification of nutritional status is inherently affected by class imbalance, as underweight adolescents typically represent a small proportion of the sample. Optimization strategies that prioritize overall accuracy, therefore, tend to show low sensitivity for minority classes, with model performance primarily determined by the correct classification of majority cases [49,50]. To address this limitation, synthetic oversampling and cost-sensitive learning have been proposed as complementary solutions. The ROSE algorithm generates synthetic minority observations from smoothed bootstrap distributions, thereby reducing the risk of overfitting associated with naive oversampling [41,43]. Cost-sensitive learning further improves minority-class detection by assigning higher penalties to misclassification errors in rare classes [51,52].

### 4.1. Class Imbalance Management and Algorithmic Performance

The imbalance between normal-weight and underweight adolescents significantly affected the baseline model’s performance, notably reducing its sensitivity for the minority class. The combined use of ROSE and cost-sensitive weighting markedly improved the detection of underweight cases while maintaining high specificity for the majority class. This result is consistent with previous methodological studies showing that strategies accounting for imbalance outperform naive optimization approaches in medical classification tasks, particularly when the prevalence of the minority class is less than 15–20% [53,54,55].

Similar trends have been observed in previous ML studies on childhood and adolescent nutrition and obesity. In these studies, models often achieved good overall discrimination but showed limited sensitivity for minority groups, such as underweight individuals or those in other high-risk categories [56]. Ensemble-based approaches, particularly RF, have often outperformed linear models and simple DTs by better capturing nonlinear relationships among predictors [57]. The lack of explicit strategies to address class imbalance in many previous studies could partly explain the reduced detection of minority nutritional categories [58].

Although the model exhibited strong discrimination, calibration analysis indicated moderate probability calibration for underweight predictions. For practical screening implementation, we recommend using the two-stage hierarchical system with the optimized threshold (0.40) rather than raw probability values.

The corrected RF model outperformed XGBoost, corroborating findings from studies on childhood obesity prediction in which uncorrected models showed low sensitivity for the minority class despite high overall discrimination metrics [59]. Notably, maintaining high specificity indicates that the applied corrections avoided overcompensation, a common limitation of naive oversampling techniques [60]. From a public health perspective, these results support the use of class-balanced performance measures rather than raw accuracy when developing tools for screening adolescent malnutrition [3,4,61].

### 4.2. Maturation-Dependent Classification Performance

The model’s performance varied across stages of biological maturation, consistent with established growth physiology. Classification was optimal in pre- and post-PHV adolescents, when growth trajectories are relatively stable, and the anthropometric distinction between nutritional categories is clear. In contrast, the decline in performance observed during the PHV phase coincided with the period of maximum interindividual variability in growth velocity and the timing of puberty [14,62,63,64].

Most prior ML studies in pediatric nutrition have relied primarily on chronological age and static anthropometric thresholds, thereby neglecting biological maturation. This age-based approach risks overlooking significant physiological heterogeneity during adolescence, particularly puberty, when growth rate and body composition change rapidly and asynchronously among individuals [65]. Ignoring these dynamic developmental factors has been shown to bias predictive models in contexts where risk profiles evolve over time [66,67].

Longitudinal growth studies show that BMI often transiently decreases during PHV, as rapid linear growth exceeds weight gain [68]. This phenomenon likely explains the reduced sensitivity to overweight observed at this stage. These results suggest that nutritional screening during puberty should incorporate an interpretation that accounts for maturation, favoring longitudinal monitoring over a one-time classification [10,16].

### 4.3. Anthropometric Dominance and Central Adiposity

Body mass has been identified as the primary predictor across all nutritional categories and maturation stages, reflecting its role in the calculation of BMI [12,13]. The model does not directly predict BMI values; rather, it classifies adolescents into IOTF-defined nutritional categories using multiple independent predictors. While body mass contributes to both BMI calculation and model input, the classification framework integrated additional variables, including waist circumference, maturation offset, behavioral measures, and anthropometric ratios. This multivariable approach identified patterns across multiple dimensions rather than reconstructing BMI from its components. The inclusion of maturation-independent predictors, such as sleep duration, physical activity, stress levels, and dietary diversity, provided information beyond that captured by age-adjusted BMI alone. Notably, waist circumference consistently ranks second, underscoring the importance of central adiposity beyond overall body size. This finding aligns with data showing that waist circumference is more strongly correlated with visceral fat and cardiometabolic risk than BMI in adolescents [69,70,71].

Previous studies have shown that adolescents with a large waist circumference have a higher prevalence of insulin resistance and dyslipidemia, even within normal BMI ranges [72,73]. In contrast, some overweight adolescents with peripheral fat distribution remain metabolically healthy [74]. These observations support the inclusion of waist circumference in screening programs for adolescents, particularly after puberty, a period of adiposity redistribution [75,76]. Traditional screening typically applies age-based BMI thresholds without accounting for maturation timing or behavioral context. Our framework provided three key advantages. First, it maintained stable classification performance across different maturation stages. Second, the model demonstrated no major misclassification between extreme categories, reducing clinically significant errors. Third, the maturation-stratified variable importance profiles revealed that predictor relevance changed substantially across PHV stages, particularly the increasing importance of age for underweight classification after PHV.

### 4.4. Age–Maturation Discordance and Underweight Identification

The increasing importance of age in classifying underweight, from the pre-pubertal to the post-pubertal period, is consistent with a growing relevance of the discrepancy between chronological age and biological maturation. Persistent underweight in older adolescents aligns with patterns of delayed biological maturation rather than transient growth variability [59,77].

Constitutional delayed growth and puberty, affecting approximately 2 to 3% of adolescents, is characterized by delayed puberty and a temporarily reduced BMI, despite normal adult development [78,79]. These adolescents often present in mid or late adolescence with underweight, despite otherwise normal health [59,79,80]. These observations highlight the need for assessment of underweight in adolescents, particularly after puberty, that accounts for maturation to ensure appropriate referral to clinical services and to avoid unnecessary interventions [59,81].

However, the model cannot definitively differentiate constitutional delay from nutritional deficiency in a cross-sectional framework, as both conditions may present with similar anthropometric profiles. On the other hand, the substantial increase in the importance of age for underweight classification from pre-PHV to post-PHV suggests that the model became increasingly sensitive to age-maturation discordance as adolescents progressed through puberty. We emphasize that our framework serves as a first-stage screening tool that flags adolescents for further evaluation rather than providing a definitive diagnosis. False positives lead to clinical review that can identify constitutional delay cases that require reassurance and monitoring, rather than true nutritional deficiency requiring intervention.

### 4.5. Behavioral Predictors and Public Health Relevance

Physical activity, sleep duration, dietary diversity, and perceived stress showed modest predictive power compared to anthropometric measures, consistent with evidence suggesting that their effects on nutritional status are primarily mediated by body composition rather than exerting direct discriminatory power in cross-sectional classification models [82,83]. The modest variable importance of behavioral predictors requires careful interpretation. Lower importance does not equate to clinical irrelevance. Body mass and waist circumference directly capture current nutritional status, whereas behavioral variables influence nutritional status through cumulative effects over time. Their modest predictive contribution in cross-sectional analyses is consistent with this temporal relationship. However, these variables provide clinically actionable information for intervention planning, and their importance patterns varied across maturation stages, suggesting stage-specific relationships that inform targeted prevention strategies. Additionally, physical activity and sleep duration influence nutritional status indirectly by modulating energy balance and growth curves. However, they ultimately affect anthropometric measures [16,18,19].

From another perspective, dietary diversity demonstrated minimal predictive value, likely due to the low granularity of food-group-based indices and their inability to capture total energy intake or macronutrient composition [39,84,85]. These measures are useful for monitoring populations, but their discriminatory value may be limited when combined with direct anthropometric indicators.

In contrast, perceived stress was the most influential behavioral predictor, contributing more to overweight classification than to underweight or normal-weight patterns. These results are consistent with work establishing a link between chronic psychosocial stress and adipose tissue accumulation via activation of the hypothalamic–pituitary–adrenal axis, cortisol-induced fat deposition, and stress-related changes in appetite and the reward system [86,87,88]. Prospective studies have shown that adolescents exposed to chronic stress have a significantly higher risk of developing overweight or obesity [89,90].

These results demonstrated that low predictive importance does not imply low clinical relevance. Behavioral factors remain essential targets for prevention and intervention strategies in public health programs, even when their contribution to algorithmic classification is modest.

### 4.6. Hierarchical Architecture and Implications for Screening

The two-stage hierarchical classification system improved the sensitivity for detecting underweight while maintaining strong discrimination between normal-weight and overweight adolescents. This structure aligns with stepwise screening paradigms commonly used in public health, in which sensitive initial identification is followed by confirmatory assessment [1,91].

Threshold optimization also demonstrated that the trade-offs between sensitivity and accuracy can be tailored to the screening context: lower thresholds are suitable for school-based surveillance, while higher thresholds are appropriate for clinical settings requiring intensive follow-up [20,23,92].

In practical screening applications, with our underweight sensitivity (0.70) and specificity (0.89), a school of 1000 students with 14% underweight prevalence would generate approximately 95 false positives while correctly identifying 98 true cases. We recommend a two-step process in which algorithmic screening triggers a confirmatory assessment by school health personnel, including verification of BMI, a review of growth charts, and a brief clinical interview. This secondary screen would identify most false positives before family notification. Family communication should emphasize routine follow-up rather than a definitive diagnosis to minimize anxiety.

Further, the trained RF model requires minimal computational resources for deployment. Prediction for 1000 students takes less than one second on standard laptop computers. The model can be implemented as a web application, spreadsheet calculator, or integrated into electronic health record systems. Data collection requires only basic anthropometric equipment and brief questionnaires that school health programs typically already administer.

### 4.7. Public Health Implications and Limitations

Overall, these results support the use of maturation-accelerating, nutritionally balanced ML models for adolescent nutritional screening. The model identifies adolescents who may benefit from further evaluation but cannot provide a definitive nutritional diagnosis. IOTF categories themselves represent screening thresholds rather than clinical diagnoses. Nutritional status assessment requires a comprehensive clinical evaluation that includes dietary intake, medical history, laboratory markers, and longitudinal growth patterns. These approaches provide balanced detection of nutritional deficiencies, avoid biases related to the accuracy of majority classes, and offer interpretable signals consistent with growth physiology.

However, several limitations must be considered. The cross-sectional design of the study does not permit assessment of longitudinal nutritional development, and maturation was estimated rather than directly observed.

The Mirwald equations yield standard errors of approximately 0.5–0.6 years, which could lead to some adolescents being misclassified into adjacent maturation categories. However, if maturation misclassification were substantial, we would expect reduced performance stability across stages. Our results showed consistent accuracy (0.823–0.839) and biologically plausible trajectories of variable importance, suggesting that measurement error is more likely to attenuate than inflate the observed patterns. Direct pubertal assessment methods, such as Tanner staging, would provide more precise classification of maturation but are rarely feasible in large-scale school settings.

Moreover, the cross-sectional design limits classification accuracy during PHV when nutritional status is most ambiguous. Longitudinal data incorporating growth velocity and repeated measurements would substantially improve performance during this critical period. Growth velocity directly indicates PHV occurrence, while short-term weight changes would differentiate transient BMI fluctuations from persistent nutritional problems.

In addition, future research should extend this maturation-accounting framework to younger, prepubertal children, using other maturation indicators, as modeling based on maximum growth velocity is not applicable before the onset of pubertal growth acceleration.

Furthermore, behavioral measures relied on subjective self-reporting, and external validation in diverse populations is required to assess the generalizability of the results. External validation in geographically and demographically distinct populations represents, indeed, an essential next step. Performance metrics may differ in populations with different nutritional epidemiology, maturation patterns, or ethnic composition. We are planning multi-site validation studies in collaboration with research teams in other North African and Middle Eastern countries to assess model generalizability across diverse populations.

Finally, body composition assessment using bioelectrical impedance or dual-energy X-ray absorptiometry would improve biological specificity by distinguishing muscle mass from adiposity. Micronutrient biomarkers can identify specific deficiencies that are not detectable by anthropometry alone. However, these measurements pose practical barriers to large-scale screening, including equipment requirements, costs, and the need for specialized training. Our approach prioritized measurements feasible for school health programs, with detailed assessment reserved for confirmed cases following initial screening.

Based on the noted shortcomings of the present study, future research should prioritize longitudinal validation, the integration of growth curves, and the evaluation of the long-term health impact of algorithm-guided screening.

## 5. Conclusions

A cost-sensitive RF model combined with ROSE yielded robust classification of adolescent nutritional status that accounted for biological maturation. The model showed balanced performance across underweight, normal weight, and overweight categories, maintained stability across maturation stages, and yielded biologically interpretable variable importance profiles.

Our findings revealed important behavioral patterns associated with nutritional status. Sleep duration increased progressively from underweight (6.5 h per night) to normal weight (6.8 h) and overweight (7.2 h) adolescents. Physical activity showed an inverse relationship with weight status, with underweight adolescents demonstrating the highest activity levels and overweight adolescents the lowest. Perceived stress increased across nutritional categories and emerged as the most influential behavioral predictor for overweight classification.

These behavioral patterns have important implications for intervention design. Overweight adolescents may benefit from integrated approaches addressing both stress management and nutritional counseling. Underweight adolescents who are physically active may require guidance on energy intake to match energy expenditure. The maturation-specific variable importance profiles suggest that intervention strategies should be tailored not only to nutritional category but also to developmental stage.

The framework is suitable for public health screening applications in schools where early identification and targeted follow-up can improve adolescent health outcomes. However, the model serves as a first-stage screening tool requiring clinical confirmation rather than providing a definitive nutritional diagnosis.

## Figures and Tables

**Table 2:** Global test performance of all machine learning models for classifying nutritional status.

Model	Accuracy	Kappa	Macro F1	Macro Sensitivity	Macro Specificity	Macro AUC	Micro AUC
Cost-sensitive RF (ROSE)	0.830	0.629	0.767	0.743	0.861	0.921	0.898
Decision Tree (rpart)	0.760	0.469	0.654	0.648	0.807	0.812	0.774
k-Nearest Neighbors (k = 5)	0.783	0.518	0.693	0.667	0.823	0.831	0.805
SVM with RBF kernel	0.822	0.616	0.758	0.743	0.860	0.902	0.872
Multinomial Logistic Regression	0.783	0.562	0.726	0.742	0.850	0.896	0.855
Extreme Gradient Boosting (XGBoost)	0.778	0.429	0.563	0.553	0.781	0.896	0.857

Abbreviations: AUC (Area Under the Curve); RBF (Radial Basis Function); RF (Random Forest); ROSE (Random Over-sampling Examples); rpart (Recursive Partitioning and Regression Tree); SVM (Support Vector Machine).

**Table 3:** Performance of Cost-Sensitive Random Forest Across Peak Height Velocity (PHV) Stages. Abbreviations: AUC (Area Under the Curve).

Model/PHV Stage	Accuracy	Kappa	Macro F1	Macro Sensitivity	Macro Specificity	Macro AUC	Micro AUC
Pre-PHV	0.827	0.701	0.814	0.815	0.895	0.936	0.919
During PHV	0.823	0.498	0.674	0.623	0.806	0.899	0.880
Post-PHV	0.839	0.646	0.777	0.760	0.870	0.931	0.908

Note. PHV = biological maturation stage. Performance metrics are based on the test dataset (*n* = 1268). Macro-AUC = mean one-vs-rest AUC across the three classes; micro-AUC = prevalence-weighted AUC.

## Data Availability

The data that support the findings of this study are available from the corresponding authors upon reasonable request. Data are stored securely, and access is granted upon completion of appropriate data-sharing agreements, in compliance with institutional and national data protection regulations.

## Supplementary Materials

The following supporting information can be downloaded at: https://www.mdpi.com/article/10.3390/nu18040660/s1, Figure S1. Two-stage hierarchical classification system diagram. Figure S2. Probability threshold optimization curves for underweight detection. Table S1. Performance metrics for the two-stage hierarchical classifier. (Stage-1 underweight detector and Stage-2 normal vs overweight classifier).
