# Supplementary material for: A Maturation-Aware Machine Learning Framework for Screening the Nutritional Status of Adolescents
Source: Nutrients. 2026 Feb 17;18(4):660. doi: 10.3390/nu18040660 (PMC12943452; doi:10.3390/nu18040660)
Supplement: Supplementary file 1 [file nutrients-18-00660-s001.zip › nutrients-4095828-supplementary.pdf]

Supplementary Materials

4.1 Supplementary Methods

S1. Detailed Class Imbalance Handling

The initial training dataset showed moderate class imbalance: normal-weight adolescents made up 68.3% of the sample, while 17.2% were overweight and 14.4% were underweight, resulting in a normal-weight to underweight ratio of approximately 5:1. This imbalance is known to hinder the detection of minority classes by classical machine learning algorithms, particularly by reducing sensitivity and recall.

To address this limitation, imbalance-correcting strategies were applied only to the training dataset, targeting different aspects of the learning process. At the data level, class imbalance was mitigated using the ROSE (Random Over-Sampling Examples) algorithm, which generates synthetic observations through a smooth bootstrap approach. ROSE was configured to increase the proportion of underweight adolescents to approximately 25% of the training observations, reducing the sampling imbalance ratio from about 5:1 to 3:1 while preserving variability across the feature space.

Independent of resampling, cost-sensitive learning was implemented in the loss function via class-specific case weighting. Underweight observations were assigned a misclassification cost 5 times higher (weight = 5) than that for normal-weight and overweight cases (weight = 1). This weighting system did not change class proportions. Still, it increased the penalty for misclassifying underweight adolescents, reflecting their greater clinical relevance and encouraging the prioritization of accurate identification of underweight while maintaining distinctions among other nutritional categories.

S1. Two-Stage Hierarchical Classification System

In addition to standard multiclass modelling, a two-stage hierarchical classification system was implemented to improve the detection of underweight adolescents. The architecture comprised two sequential Random Forest models designed to prioritize the identification of underweight adolescents without compromising performance for the majority of classes.

The first-stage function served as a binary underweight detector, trained on ROSE-augmented data and employing cost-sensitive learning with higher misclassification penalties for underweight observations. This combination of data-level resampling and loss function weighting enabled the learning of underweight-specific patterns while explicitly penalizing false negatives. Adolescents whose predicted underweight probability exceeded the predefined threshold (0.40) were classified as underweight at this stage.

The second stage was applied only to adolescents classified as not underweight by the first stage and distinguished between normal weight and overweight categories using a standard Random Forest model trained without oversampling or class weighting. By decoupling underweight detection from normal-weight and overweight classification, this hierarchical structure enabled early identification of underweight adolescents while maintaining robust discrimination among the main nutritional categories.

https://doi.org/10.3390/nu18040660

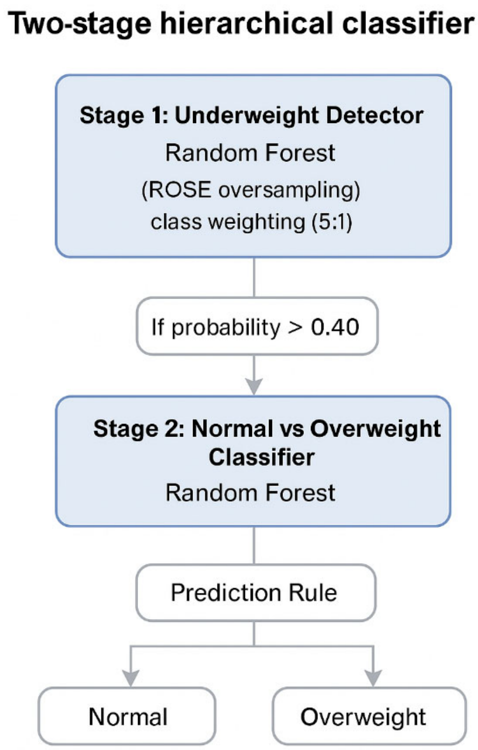

Figure S1. Two-stage hierarchical classification system diagram.

S2. Probability Threshold Optimization

Post-training probability threshold optimization was conducted to refine the trade-off between sensitivity and accuracy for the first-stage underweight detector. Thresholds from 0.30 to 0.80, in increments of 0.02, were evaluated. For each threshold, accuracy, recall, F1 score, and the number of misclassifications were calculated. Three optimization strategies were compared: (i) maximizing the F1 score, (ii) prioritizing high accuracy (≥ 0.85), and (iii) maximizing the product of accuracy and recall, in line with approaches recommended for threshold selection in clinically unbalanced classification problems. The final threshold (0.40) was selected to achieve a clinically balanced compromise between sensitivity and accuracy.

https://doi.org/10.3390/nu18040660

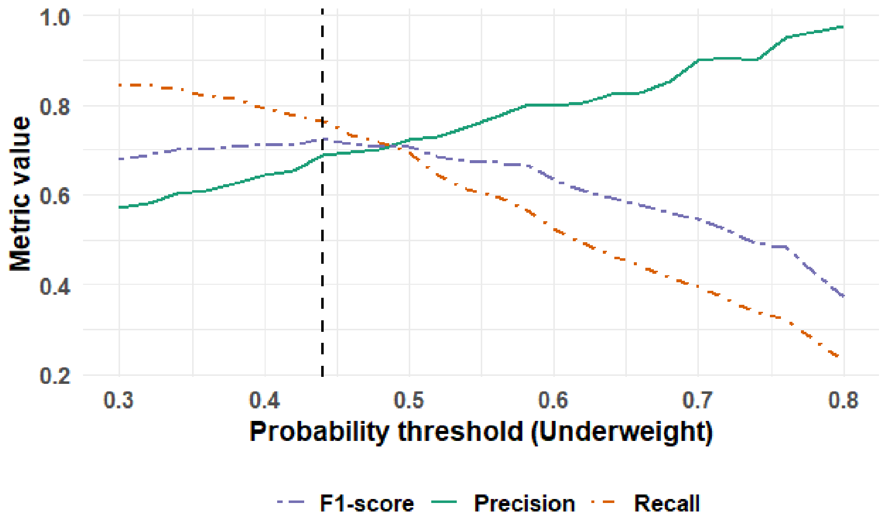

Strategy	Threshold	Precision	Recall	F1
Max F1-score	0.44	0.690	0.765	0.725
High Precision (>=0.85)	0.68	0.854	0.415	0.559
Max Precision×Recall	0.44	0.690	0.765	0.725

Figure S2. Probability threshold optimization curves for underweight detection.

4.2. Supplementary Results

Threshold Optimization

Figure S2 shows the evolution of accuracy, recall, and F1 score as a function of probability thresholds for predicting underweight. As expected, increasing the threshold improved accuracy but reduced recall, reflecting the classic accuracy-recall trade-off. The F1 score peaked at a threshold of 0.44, indicating the most balanced trade-off between sensitivity and accuracy (accuracy = 0.69; recall = 0.77; F1 = 0.73).

A more conservative strategy, prioritizing accuracy, identified a higher threshold (0.68), achieving very high accuracy (0.85) at the cost of significantly reduced recall (0.42). These results suggest using thresholds of approximately 0.40–0.45 for screening applications, whereas higher thresholds may be preferable in confirmatory or diagnostic settings, where minimizing false positives is critical.

Supplementary Tables

Table S1 summarizes the diagnostic performance of the two-step hierarchical classification system. In the first step, the underweight detector achieved an accuracy of 0.871, with strong discriminative performance (AUC = 0.911) and high sensitivity for underweight adolescents (0.732). Accuracy was intentionally reduced (0.538) to optimize sensitivity and minimize the number of undetected cases of underweight.

In the second step, classification between normal-weight and overweight adolescents resulted in superior overall performance, with an accuracy of 0.907 and an AUC of 0.938. The classifier demonstrated excellent specificity (0.972) and high accuracy (0.855) for identifying overweight, as well as acceptable sensitivity (0.647). These results indicate that the hierarchical architecture effectively prioritizes the early detection of underweight adolescents while maintaining high classification accuracy for other nutritional categories.

https://doi.org/10.3390/nu18040660

Table S1. Performance metrics for the two-stage hierarchical classifier. *(Stage-1 underweight detector and Stage-2 normal vs overweight classifier).*

Metric	Stage-1 Underweight Detector	Stage-2 Normal vs Overweight Classifier
**Accuracy**	0.871	0.907
**Kappa**	0.545	0.681
**Sensitivity**	0.732	0.647
**Specificity**	0.894	0.972
**Precision**	0.538	0.855
**F1-score**	0.620	0.736
**AUC**	0.911	0.938

https://doi.org/10.3390/nu18040660
